# Assays Used for Discovering Small Molecule Inhibitors of YAP Activity in Cancers

**DOI:** 10.3390/cancers14041029

**Published:** 2022-02-17

**Authors:** Subhajit Maity, Artem Gridnev, Jyoti R. Misra

**Affiliations:** Department of Biological Sciences, University of Texas at Dallas, Richardson, TX 75080, USA; subhajit.maity@utdallas.edu (S.M.); artem.gridnev@utdallas.edu (A.G.)

**Keywords:** Hippo signaling, cancer, drug discovery, high throughput screening

## Abstract

**Simple Summary:**

Cancer is a disease in which cells grow in an uncontrolled manner. This can be due to excessive cell proliferation or reduced cell death or a combination of the two. The Hippo signaling pathway, when misregulated, promotes excessive growth and cancer development by inducing uncontrolled cell proliferation and inhibiting cell death. This is achieved due to unregulated activity of the oncogenic effector of this pathway, YAP/TAZ. Therefore, it is critical to develop inhibitors to disrupt YAP activity in cancers. This article reviews the different types of assays that are used in development of small molecule inhibitors for YAP activity in cancers.

**Abstract:**

YAP/TAZ are transcriptional coactivators that function as the key downstream effectors of Hippo signaling. They are commonly misregulated in most human cancers, which exhibit a higher level of expression and nuclear localization of YAP/TAZ, and display addiction to YAP-dependent transcription. In the nucleus, these coactivators associate with TEA domain transcription factors (TEAD1-4) to regulate the expression of genes that promote cell proliferation and inhibit cell death. Together, this results in an excessive growth of the cancerous tissue. Further, YAP/TAZ play a critical role in tumor metastasis and chemotherapy resistance by promoting cancer stem cell fate. Furthermore, they affect tumor immunity by promoting the expression of PD-L1. Thus, YAP plays an important role in multiple aspects of cancer biology and thus, provides a critical target for cancer therapy. Here we discuss various assays that are used for conducting high-throughput screens of small molecule libraries for hit identification, and subsequent hit validation for successful discovery of potent inhibitors of YAP-transcriptional activity. Furthermore, we describe the advantages and limitations of these assays.

## 1. Introduction

**Figure 1:**
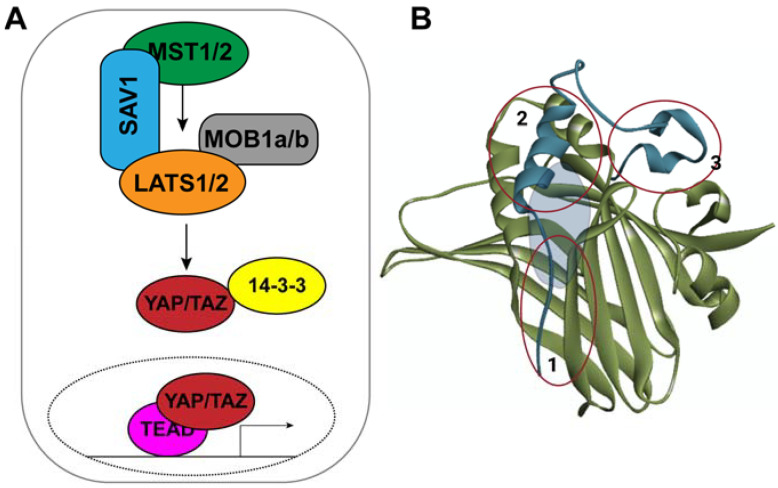
(**A**) Simplified schematic of the Hippo signaling pathway. MST1/2 phosphorylates LATS1/2, which in turn phosphorylates YAP/TAZ. Phosphorylated TAP/TAZ is retained in the cytoplasm by the 14-3-3 proteins. (**B**) Structure of TEAD (green) and YAP (blue) showing the 3 interfaces of interaction and the central hydrophobic pocket in grey.

The Hippo signaling pathway plays a central role in regulation of cell proliferation, cell death and cell fate determination. It consists of a kinase cascade module that negatively regulates the oncogenic nuclear activity of a transcriptional coactivator (Figure 1A) [1]. The kinase cascade module is composed of the serine threonine kinases MST1/2 and the large tumor suppressor (LATS1/2) and their respective obligate cofactors SAV1 and MOB1/2. MST1/2 phosphorylates and activates LATS1/2, which in turn phosphorylates YAP/TAZ. Phosphorylated YAP/TAZ becomes sequestered in the cytoplasm by interacting with 14-3-3 proteins. Hypo/unphosphorylated YAP/TAZ translocate into the nucleus and associate with TEAD1-4 transcription factors to regulate the expression of a large number of target genes [2,3]. Many of the YAP target genes encode matricellular proteins that promote cell proliferation. Other target genes inhibit apoptosis. Upregulation of these genes together leads to tissue overgrowth. YAP plays a central role in organ size regulation during embryonic development and seems dispensable in most organs in adults.

YAP is commonly misregulated in most human cancers; where downstream of various oncogenic signaling pathways, YAP/TAZ are overexpressed. In many cancers, the overexpressed YAP/TAZ localize to the nucleus [4,5]. Overexpressed YAP/TAZ undergo phase separation and create transcriptional hubs at the super-enhancers, causing sustained high-level expression of genes that promote cell proliferation and inhibit apoptosis [6,7]. Many of these cancers exhibit addiction to YAP-dependent transcription and are susceptible to YAP inhibition [8]. Furthermore, YAP additionally promotes the maintenance of cancer stem cell fate and promotes chemotherapy resistance [9,10]. Moreover, YAP interferes with anti-tumor immunity by promoting the expression of PD-L1 [9,11,12,13]. Thus, YAP plays a central role in regulating multiple aspects of cancer development and thus, provides a critical point for therapeutic intervention. Furthermore, given that YAP function is dispensable for most adult organs, it is an attractive target for cancer therapy. In addition to cancer, YAP/TAZ are also involved in fibrosis, where they play an important role in the conversion of fibroblasts into contractile myofibroblasts, which secrete excessive amounts of collagen and other connective tissue components. Inhibiting YAP has been shown to improve experimentally-induced fibrosis in animals. Therefore, it is highly imperative to develop small molecule inhibitors of YAP activity for treatment of YAP-dependent cancers and fibrosis.

YAP primarily regulates the expression of its target genes by associating with TEAD1–4 transcription factors on one hand, and components of the basal transcription machinery on the other. Thus, YAP activity can be inhibited by the molecules that disrupt these interactions. However, YAP is an intrinsically disordered protein, and the basal transcription machinery components with which it interacts remain unknown. On the other hand, its interaction with TEAD is well studied. Structural studies revealed that YAP binds to TEAD tightly by engaging the N-terminal 100 amino acids, and this interaction is mediated by three interfaces (Figure 1B) [14,15,16,17,18]. The first interface is between a beta sheet of YAP and that of TEAD. The second interface consists of an alpha helix of YAP and two alpha helices of TEAD. The third interface is mediated by the amino acids 85–100 of YAP, which is referred to as the “omega loop”, and this interface contributes toward the maximum free energy of YAP–TEAD binding. Mutations in several of the key residues in this region completely disrupt YAP–TEAD interaction. However, this interface is very broad and shallow and is thus not easily amenable to interference by small-molecule inhibitors. Therefore, current efforts are directed toward the development of molecules that destabilize TEAD and allosterically inhibit its interaction with YAP.

One of the unique features of TEAD transcription factors is that they undergo autopalmitoylation. The palmitic acid is covalently conjugated to a conserved cysteine residue and occupies a central hydrophobic pocket [19,20]. Palmitoylation is required for TEAD stability and small molecules that occupy this pocket prevent TEAD palmitoylation and render the molecule unstable [21,22]. Furthermore, they allosterically inhibit YAP–TEAD interaction. Since the central hydrophobic pocket is the most druggable site, currently, significant effort is aimed at developing potent inhibitors that bind to this site. Furthermore, this site is highly conserved among the four TEAD isoforms. Therefore, it is possible to identify pan-TEAD inhibitors that bind to this region. Interestingly, a number of investigational compounds are available that bind to this pocket. However, no compound has entered clinical trials yet. It is, therefore, important to develop new chemotypes that can bind to this site and inhibit all TEAD isoforms.

Small molecule inhibitors are a mainstay of cancer therapy. Traditionally, identification of hit compounds relied on large-scale unbiased high-throughput screens of chemical libraries, conducted with the help of automated robotic systems. Such screens are expensive, time consuming, and require specialized equipment. Recently, advances in *in silico* prediction methods and the availability of affordable computational resources have prompted a common practice: to conduct ultra large virtual ligand screening, where more than 1 billion small molecules can be computationally screened to predict the ones, which are likely to bind to a target protein [23]. The use of deep learning methods has further revolutionized such ultra large screens and has enabled isolation of potent and specific inhibitors by experimentally screening a relatively manageable number of compounds. Similarly, computational approaches such as V-SYNTHES conduct large scale fragment-based drug development, providing another path to discover and optimize potent inhibitors [24]. However, such computational methods are applicable only for proteins for which structural information is available. Given the availability of high-quality crystallographic structures for TEAD, these kinds of approaches to develop inhibitors of TEAD are more feasible. For targets such as YAP, for which no structure is available, and the knowledge about other interactors are limited, unbiased experimental screens will continue to remain the method of choice for the identification of inhibitors.

Whether one intends to conduct a traditional unbiased high-throughput screen or a relatively less intensive small library screen, following a virtual ligand screening campaign, utmost care must be exercised to design assays for successful identification of hit compounds and subsequent validation and improvement through medicinal chemistry. Here we describe different assays that are used for the successful screening of small molecule libraries for the identification and subsequent validation of YAP activity inhibitors.

## 2. Assays for High Throughput Screening

### 2.1. Cell Culture-Based Transcriptional Reporter-Based Assays

**Figure 2:**
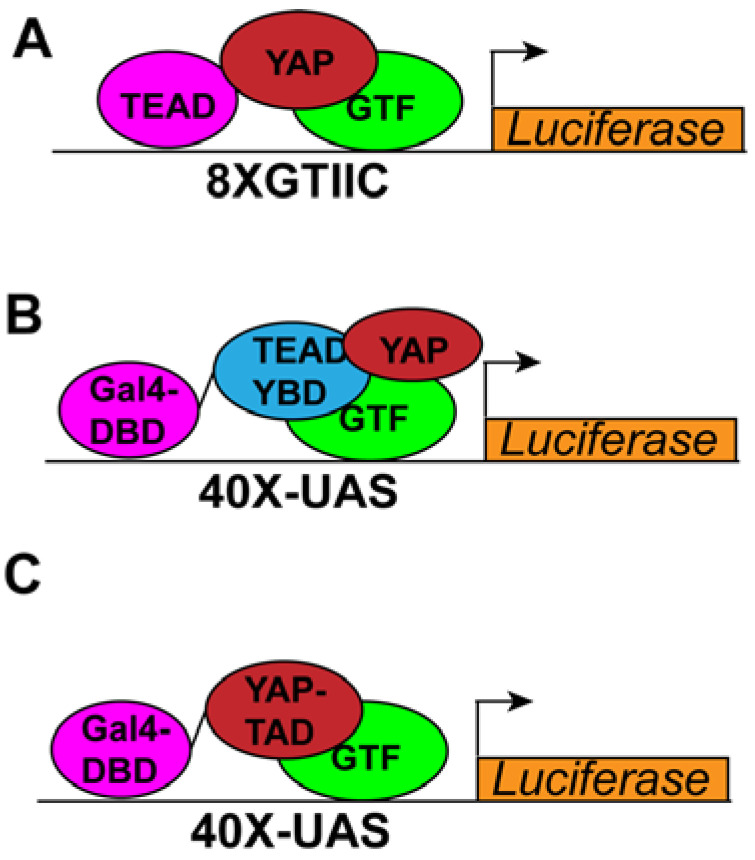
Schematics of transcriptional reporter assay with (**A**) Fluc fused to 8XGTIIC sequence. (**B**) Fluc fused to UAS sequence and TEAD–YBD fused to GAL4-DBD and (**C**) Fluc fused to UAS sequence and YAP-TAD fused to GAL4-DBD.

Most high-throughput assays for screening small molecule libraries use cell culture-based transcriptional reporter assays, where a firefly luciferase (Fluc) is under the control of a multimerized copy of the TEAD binding site from the SV40 enhancer region (8XGTIIC) (Figure 2A) [25]. When YAP is activated, luciferase expression is increased. As a control, the cells express *Renilla* luciferase (Rluc) from a constitutive promoter. This is used to normalize the firefly luciferase levels, and compounds that inhibit transcription in general, or are cytotoxic, affect the expression of Rluc. There are several variations of this assay that use multimerized TEAD binding sites from the CTGF or CYR61 promoter regions. The basal activity of the 8XGTIIC-Fluc reporter is very low when the cells are grown at high density, on hard surfaces, and in the presence of serum. This reporter activity can be increased by several means such as hydrogen peroxide treatment or treatment with the MST1/2 inhibitor XMU-MP1 [26]. We have seen that co-transfecting the cells with a plasmid encoding non-phosphorylatable YAP (YAP-5SA) or TAZ (TAZ S89A) dramatically increases the reporter activity and helps to identify inhibitors. Compounds that bind to TEAD inhibit the reporter activity in presence of both YAP-5SA and TAZ-S89A. An advantage of this assay system is that compounds that are cytotoxic can be readily eliminated. One of the limitations of this transcriptional reporter is that it uses the endogenous TEAD and does not discriminate between the TEAD isoforms. 

Another widely used cell culture-based transcriptional reporter assay makes use of the binary UAS-GAL4 system. In this system, the Fluc is under the control of the UAS sequence and the YAP-binding domain (YBD) of different TEAD isoforms is fused to the DNA-binding domain (DBD) of the yeast GAL4 transcription factor (Figure 2B) [27]. The GAL4-DBD–TEAD–YBD fusion proteins bind to the UAS sequence and upregulate expression of Fluc. As is the case with the assay described above, constitutively expressed Rluc is used to normalize the Fluc expression. Verteporfin was discovered using this assay. One of the advantages of this system is that it helps to distinguish if a given compound inhibits a specific isoform of TEAD. However, we have observed that Fluc expression from this reporter is very high, and even a compound that exhibits a strong inhibition in the previous assay may exhibit only moderate inhibition in this assay. Therefore, one must be cautious in discarding compounds as false negatives when using this assay. Another drawback of this reporter is that many compounds may interfere with binding of GAL4-DBD with UAS sequences. Such compounds may appear as positive and should be independently tested in the 8X-GTIIC-Fluc reporter system to ensure that they specifically inhibit YAP activity.

Another variation of the GAL4-based assay is that it can be used to isolate compounds that specifically inhibit YAP-transcriptional activity. Since YAP can also interact with transcription factors other than TEAD1–4, TEAD inhibitors may not completely block YAP activity [2,3]. It is, therefore, desirable to develop compounds to specifically inhibit interaction of the YAP-transcriptional activation domain (TAD) with its cognate partners. To screen for such compounds, the YAP-TAD can be fused to the GAL4-DBD and can be used along with the UAS-Fluc reporter system. Recently, it was reported that amino acids 450–504 in the YAP-TAD (TAD short or TADs) exhibit almost the same transcriptional activity as the full-length TAD [28]. Thus, one can also use the GAL4-DBD-TADs along with the UAS-Fluc reporter to screen for compounds that inhibit the interaction of this region, thus inhibiting YAP-transcriptional activity (Figure 2C). In general, UAS-GAL4-based reporter assays are so robust that most inhibitors exhibit only moderate effect. Therefore, using one of the available investigational compounds as a positive control will help determine the cutoff limit to prevent eliminating compounds as false negatives.

In all of these assays, one should observe that some compounds may simply inhibit Fluc activity, which will appear as genuine positives. Therefore, care must be exercised to test the compound in cells expressing Fluc from a constitutive promoter. Additionally, one should also check if they have general cytotoxicity by parallelly conducting a CellTiter-Glo™ assay, which measures the general health of cells using ATP production as a proxy. Also, one can examine if a compound belongs to the PAINS group to generally cause cytotoxicity [29].

### 2.2. Assay for Screening YAP–TEAD Interaction Inhibitors

**Figure 3:**
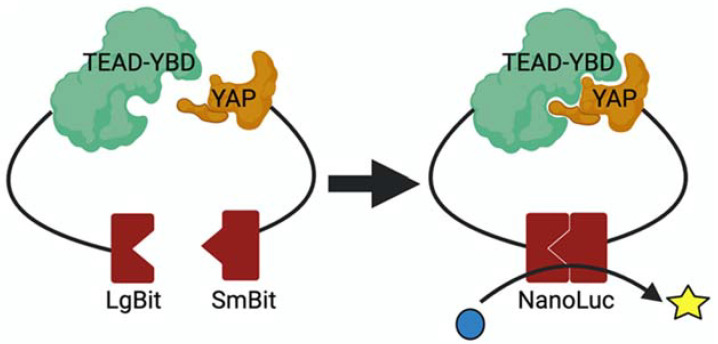
NanoLuc based bioluminescence assay. NanoLuc fragments, LgBit and Smbit are fused to TEAD and YAP respectively. Interaction between TEAD and YAP reconstitutes NanoLuc activity.

For screening compounds that inhibit YAP–TEAD interaction, a recently developed assay based on NanoLuciferase (NanoLuc) bioluminescence complementation can be used [30]. NanoLuc is an engineered small and bright luciferase, which can be split into a small fragment (SmBit) and a large fragment (LgBit). These two fragments do not interact with each other. However, when fused to two different proteins that interact with each other, interaction between the two proteins brings these fragments in close proximity of each other and reconstitutes the NanoLuc enzymatic activity, which can be quantified by measuring luminescence in a plate reader (Figure 3). 

For screening YAP–TEAD interaction inhibitors, the TEAD binding region of YAP is fused to SmBit and the TEAD–YBD is fused to LgBit [31]. These fusion proteins can be easily expressed and purified from E. coli. As expected, on their own, these proteins do not have any NanoLuc enzymatic activity. However, when equimolar quantities of these proteins are mixed with each other, a dramatic increase in luminescence is observed. For screening compounds that disrupt YAP–TEAD interaction, the compound is incubated with LgBit-TEAD–YBD overnight and the next morning, and an equimolar amount of SmBit–YAP is added for 5 min and the NanoLuc activity is measured. Vehicle alone should be used as a negative control, and compounds such as VT107 should be used as positive control. Celastrol was identified to inhibit YAP–TEAD interaction using this method. The advantage of this assay is that one can screen compounds at relatively higher concentrations compared to cell culture-based assays, as it can tolerate what would otherwise be cytotoxic. Furthermore, because it can tolerate cytotoxic compounds, and these can later be modified to render them nontoxic. One of the limitations of this assay is that certain compounds can undergo colloidal aggregation or denature the proteins and will appear as positives. Moreover, compounds that simply inhibit the interaction between SmBit and LgBit or inhibit NanoLuc activity will also appear as positive. Therefore, caution must be exercised to rule out these possibilities by testing them in other assays such as coimmunoprecipitation. These fusion proteins can also be expressed in cells to directly test compounds in cell culture.

### 2.3. Assay for Screening Covalent TEAD Palmitoylation Inhibitors

Since the palmitic acid is covalently linked to a conserved cysteine residue in TEAD, one can screen for inhibitors that form a covalent bond with the cysteines by using a thiol-reactive pro-fluorescent probe such as N-(4-(7-diethylamino-4-methylcoumarin-3-yl)phenyl)maleimide (CPM) [32]. Because of the maleimide substitution on the phenyl group that modulates the resonance between the coumarin carbonyl and 7-amino groups, normally the fluorescence in CPM is quenched. However, upon reaction with a thiol, CPM fluorescence increases dramatically. Any compound that prevents this conjugation will decrease the CPM fluorescence. This has been successfully used for screening of covalent inhibitors of TEAD to identify kojic acid analogs as covalent TEAD inhibitors. 

### 2.4. Fluorescence Polarization Assay

This a commonly used method to study binding kinetics between a drug molecule and the target protein. A well-established FP assay is available for studying small molecule inhibitors of YAP–TEAD interaction [33]. This uses a fluorescently-labelled YAP-derived small peptide that binds to TEAD with high affinity. Any compound that interferes with YAP–TEAD interaction causes a decrease in fluorescence polarization. Whereas this assay has not been used for large scale screens, it can easily be used for this purpose.

## 3. Assays for Hit Validation

### 3.1. Effect on YAP-Target Gene Expression

Once a hit compound is identified, it must be tested to assess whether it affects YAP-target gene expression. To do that, cancer cell lines such as MDA-MB-231 that harbor mutations in the Hippo pathway are treated with different doses of the compound, and the RNA is extracted and converted to cDNA. Subsequently, using gene specific primers, the expression of target genes such as CTGF, AREG, AXL is determined by quantitative RT-PCR. House-keeping genes such as GAPDH, actin, or tubulin are used for normalization. To quantitatively interrogate gene expression changes at the genome-wide scale, the whole transcriptome can be analyzed by RNA seq.

### 3.2. YAP and TEAD Localization

Certain Compounds can inhibit YAP-transcriptional activity simply by inhibiting the nuclear translocation of YAP and TEAD. Therefore, it is necessary to verify if a compound affects localization of YAP and/or TEAD. This can be performed by treating cells with vehicle or the drug compound and staining them with a YAP and pan-TEAD specific antibodies. The nuclear-to-cytoplasmic ratio of the drug treatment condition relative to the vehicle treated controls reflects any change in the localization of the proteins induced by the drug.

### 3.3. TEAD Stability Assay

TEADs undergo covalent modification with palmitic acid, and compounds that bind to the central hydrophobic pocket of TEADs and prevent their palmitoylation, render them unstable [19,20,21,22]. To examine if an inhibitor affects endogenous TEAD stability, cells are either treated with vehicle alone or different doses of a drug compound overnight and subsequently, the cell lysate is examined for the amount of TEAD present by Western blotting. TEAD quantities are normalized to a number of house-keeping genes like actin, tubulin, or GAPDH. It is possible that a compound can affect TEAD transcription. Therefore, to rule out this possibility, one should check TEAD transcript levels by quantitative RT-PCR. It is also common practice to stop new protein synthesis by simultaneous treatment with cycloheximide and examine how the drug affects TEAD stability. One can also examine if a compound affects the stability of a specific TEAD isoform by transfecting cells with plasmids expressing epitope-tagged TEAD isoforms. In this case, one should be cautious to ensure that the drug does not affect TEAD expression from the plasmid. A plasmid expressing GFP from the same promoter that regulates TEAD expression can be co-transfected along with the plasmid expressing epitope-tagged TEAD. Absence of any effect on GFP expression levels would suggest a lack of any apparent effect of the compound on TEAD expression from the plasmid. 

### 3.4. TEAD Palmitoylation Assay

**Figure 4:**
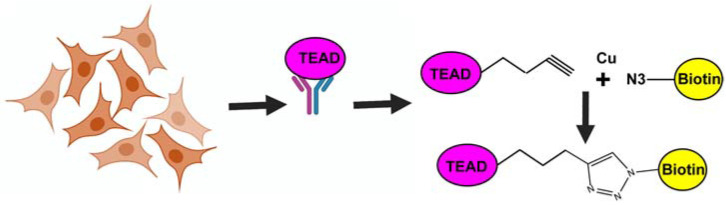
Schematic showing TEAD palmitoylation assay. Cells expressing TEAD are incubated with alkyne palmitate. Immunoprecipitated TEAD is covalently linked to biotin azide by click chemistry. Biotin is detected with fluorescently labeled streptavidin.

If a compound directly binds to the TEAD central pocket and inhibits palmitoylation, it can be examined by using cell culture or purified proteins. In cell culture, cells transfected with epitope-tagged TEAD are incubated with alkyne palmitic acid along with vehicle or the drug compound. This allows metabolic labeling of the TEADs with the alkyne palmitic acid. Subsequently, TEAD is immunoprecipitated and the covalently attached alkyne palmitic acid is conjugated with azide-biotin using copper catalyzed click chemistry (Figure 4). Then the biotin conjugated palmitic acid is detected by Western blotting, using a fluorescently-tagged Streptavidin. In parallel, the total TEAD level is determined using a TEAD specific antibody. The ratio of the streptavidin signal to the total TEAD levels are used to determine if a compound inhibits TEAD palmitoylation. In case of a vehicle-treated sample, a strong palmitoylation signal is detected. As a positive control, one can use VT107, which has been shown to robustly inhibit TEAD palmitoylation [22].

One can also perform a variation of this assay using purified recombinant protein to examine if a compound can inhibit TEAD palmitoylation. All TEAD isoforms can be easily expressed and purified from *E. coli*. However, these proteins purified from *E. coli* are also palmitoylated. Therefore, they need to be de-palmitoylated by treating with hydroxylamine, which cleaves the thioester linkage between the palmitic acid and the cysteine on TEAD. The de-palmitoylated protein is then incubated with alkyne palmitic acid to re-palmitoylate it in presence of vehicle or a drug. The re-palmitoylated protein can be conjugated to biotin-azide by click chemistry and detected by fluorescently labeled streptavidin as describe above.

### 3.5. Assay for Confirming YAP–TEAD Interaction Inhibitors

If a compound significantly disrupts the interaction between YAP and TEAD, this can be confirmed by a co-immunoprecipitation assay. This assay can be performed using endogenous YAP and TEAD using antibodies against these proteins or using cells transfected with epitope-tagged YAP and TEAD, in which case, the antibodies against the epitope tags can be used. Cells are either treated with vehicle alone or different doses of the compound, and either YAP or TEAD is immunoprecipitated, and the immune complexes are examined for the amount of the other protein using Western blotting. Since many drugs that bind to TEAD can induce its degradation, presence of a lower amount of TEAD could result from the degradative effect of a compound. For this reason, one should pull down YAP, as most drugs do not seem to affect YAP stability.

### 3.6. Assays for Testing Direct Binding of Inhibitors with TEAD

#### 3.6.1. Differential Scanning Fluorimetry (DSF)

This assay is used post screening to determine target engagement, and to detect thermally-induced protein denaturation by measuring the fluorescence of a compound that preferentially binds to unfolded proteins [34]. SYPRO Orange is a commonly used dye that binds to hydrophobic regions exposed by thermal unfolding of proteins. This is also known as the thermal shift assay, as it can be used to determine the “shift”, or difference in melting temperature between untreated and compound-treated protein. In this assay, purified recombinant TEAD proteins are incubated with the compound and SYPRO Orange. Subsequently, the mixture is subjected to a temperature gradient, where the temperature is gradually increased. The drug-bound protein becomes denatured at a higher temperature compared to the unbound protein. The denaturation of the protein can be observed indirectly by an increase in fluorescence from the dye, which binds to denatured protein. This can be performed in a simple qRT-PCR machine. A variation of DSF called nano DSF uses the intrinsic fluorescence of tryptophan and tyrosine of the protein as a function of temperature.

#### 3.6.2. Cellular Thermal Stability Assay (CETSA)

This assay is used in cells for examining target engagement with the protein in cells, and is based on the same principle as DSF: that binding of a ligand to the receptor protein renders it resistant to thermal denaturation [35]. In this assay, cells are treated with vehicle alone or with the compound of interest. Subsequently, the cells are lysed and centrifuged and the supernatant containing TEAD is subjected to heating in a temperature gradient. Then, the sample is centrifuged at a high speed to separate the denatured and nondenatured protein. The soluble fraction is then examined for the amount of TEAD by western blotting. If a compound binds to TEAD, the protein will denature at a higher temperature compared to the vehicle-treated sample.

#### 3.6.3. Isothermal Titration Calorimetry (ITC)

ITC is a highly sensitive quantitative method used to determine the thermodynamic parameters of interaction between the drug and target in solution. It is used to determine the binding affinity (association constant, K_a_), and changes in enthalpy and binding stoichiometry [36]. This assay uses a sensitive calorimeter that consists of two chambers contained in an adiabatic jacket. In the sample cell, the small molecule is added to the purified recombinant TEAD protein in precisely measured small aliquots, while the control cell contains the protein in buffer. If the small molecule binds to TEAD, the small amount of binding energy is released as heat, which changes the temperature in the sample cell with respect to the reference cell. The heat released (enthalpy) can be measured from the amount of electrical energy input needed to keep the reference cell in thermal equilibrium with the sample cell. From the measured enthalpy, Gibbs free energy, entropy, association constant, and binding stoichiometry values are calculated. 

#### 3.6.4. Surface Plasmon Resonance (SPR) 

This is an optical technique used to determine the kinetics of protein–drug interaction, based on changes in the refractive index near a metal surface [37]. Here, the target protein is immobilized on a sensor chip coated with gold foil. Binding of the ligand to the target protein causes an increase in mass and dissociation of the ligand causes a decrease in mass. These mass changes in turn affect the refractive index on the sample side of the chip. When illuminated with light of a specific wavelength at a precise angle, electron waves are produced on the metal surface. This is called surface plasmon resonance (SPR). When the drug molecule binds to the immobilized target protein, the SPR pattern is changed and a measurable difference in emitted energy is observed. Thus, biotinylated TEAD can be immobilized on the sensor chip and the drug molecule is allowed to bind to TEAD to determine the kinetics of binding.

#### 3.6.5. Binding Mode Analysis

To determine the binding mode, the compound can be co-crystallized with different TEAD isoforms, and the crystallographic structure provides information about the binding mode. Alternatively, one can use HSQC NMR spectroscopy [38]. In this case, the binding of the compound perturbs the environment of the amino acids that interact with the drug molecule and broadens the spectral lines corresponding to these amino acids.

### 3.7. Functional Assays to Detect Effect of Small Molecule Inhibitors on Cancer 

#### 3.7.1. Clonogenic Assay

This assay is commonly employed to test the ability of a compound to inhibit growth of cancer cells [39]. In this assay, a given number of cells (~100–200) are plated in media containing vehicle alone or different doses of the drug and allowed to grow as individual colonies. After several weeks, when visible colonies can be seen, the cells are fixed and stained with crystal violet dissolved in methanol. Then, the number of colonies is counted to assess the drug concentration that inhibits the ability of the cells to form large colonies.

#### 3.7.2. MTT Assay

This is a quantitative colorimetric assay that is used to measure cellular metabolic activity as an indicator of cell viability, cell proliferation, cytotoxicity, or cytostatic activity [40]. This assay is based on the reduction of the yellow tetrazolium salt MTT (3-(4,5-dimethylthiazol-2-yl)-2,5-diphenyltetrazolium bromide or) to formazan crystals by metabolically active cells via NAD(P)H-dependent oxidoreductase enzymes. The insoluble formazan crystals are dissolved using a solubilization buffer and the resulting, purple-colored solution is quantified by measuring absorbance in a spectrophotometer. The higher the intensity of the solution, the greater the number of viable, metabolically active cells. Compounds that inhibit YAP activity will inhibit cell proliferation, and will therefore exhibit less formazan crystal formation.

#### 3.7.3. Inhibition of Tumor Growth in Mouse Models of Cancer

After a hit compound is found to inhibit the YAP-transcriptional reporter, target gene expression, and growth in cell culture, it must ultimately be tested for its ability to inhibit tumor growth in animal models. Most commonly, mouse xenograft models are used, where cancer cells are transplanted subcutaneously in nude mice and treated with vehicle alone or different doses of a compound for several weeks and the growth of the tumor is measured at regular intervals [41]. Cancer cells stably expressing luciferase can be imaged *in situ* using bioluminescence-based small animal imaging systems. If a mouse genetic model of the cancer is available, the drug should be tested in the model to examine if it can inhibit tumor growth and metastasis in vivo.

## 4. Conclusions

The Hippo pathway effectors, YAP/TAZ are commonly misregulated in most human cancers and play a critical role in tumor growth, metastasis, immunity, and therapy resistance. Thus, they provide a critical point for therapeutic intervention for many cancers. YAP/TAZ exert their transcriptional activity by binding to TEAD1–4. Since YAP/TAZ are intrinsically unfolded proteins and lack definitive structure, current efforts are aimed toward indirectly inhibiting YAP activity by inhibiting TEAD palmitoylation and stability. Recently, the use of artificial intelligence has led to significant progress in structure-based *in silico* virtual ligand screening as well as fragment-based ligand design. However, while such methods are suitable for developing TEAD inhibitors, development of true YAP inhibitors will require unbiased high-throughput screens of protein–protein interaction (PPI) inhibitors. Interestingly, artificial intelligence has been used to design many such PPI inhibitor libraries. The assays outlined in this review will provide a framework for designing high-throughput assays for screening small molecule libraries and subsequent validation of the hit compounds.
